# Supplementary material for: A study of systemic risk spillovers in Asian emerging markets and Chinese stock market
Source: PLoS One. 2025 May 5;20(5):e0322381. doi: 10.1371/journal.pone.0322381 (PMC12052167; doi:10.1371/journal.pone.0322381)
Supplement: Attachment — Submitted filename: review comments.docx [file pone.0322381.s001.docx]

**A Study of Systemic Risk Spillovers in Asian Emerging Markets and Chinese Stock Market**

**Review Comments**

1) Abstract Clarity: The abstract contains minor phrasing issues. For example, the sentence “the study fills a gap in understanding the asymmetric across these markets” is unclear and should be revised for better clarity and readability.
2) Dataset selection: The rationale for market selection aligns with MSCI classifications, but the exclusion of certain regional markets should be more clearly justified.
3) Risk Spillover Dynamics: While the study analyzes risk spillovers broadly, it would benefit from examining how risks propagate during specific financial crises. Although global events such as COVID-19 and the Russia-Ukraine war are mentioned, their specific impacts on systemic risk dynamics require more detailed exploration.
4) Literature and Emerging Markets: The literature review could be expanded to include recent studies on systemic risk and financial contagion, particularly in emerging markets.
5) Visual Aids: Incorporating visual aids like influence networks to illustrate risk spillovers would improve the clarity and accessibility of the findings.
6) Policy Recommendations: While the study suggests strengthening macroprudential frameworks, the recommendations are generic. The findings should be used to propose specific policies to mitigate systemic vulnerabilities in the region.
7) Typographical Errors: The paper includes typographical errors, such as inconsistencies in notations. For instance, the price at day $t$ is referred to as $R_{t}$ in the text but is denoted as $P_{t}$ in the equations.
