# Supplementary material for: A study of systemic risk spillovers in Asian emerging markets and Chinese stock market
Source: PLoS One. 2025 May 5;20(5):e0322381. doi: 10.1371/journal.pone.0322381 (PMC12052167; doi:10.1371/journal.pone.0322381)
Supplement: Attachment — Submitted filename: Response to Reviewers 2.docx [file pone.0322381.s003.docx]

**A Study of Systemic Risk Spillovers in Asian Emerging Markets and Chinese Stock Market**

**Review Comments**

Reviewer #2:

1) Abstract Clarity: The abstract contains minor phrasing issues. For example, the sentence “the study fills a gap in understanding the asymmetric across these markets” is unclear and should be revised for better clarity and readability.

A: This study examines systemic risk spillover effects between China’s Shanghai Stock Exchange (SSE) and seven Asian emerging markets within the context of increasing global financial integration. Utilizing Quantile Regression and Conditional Value-at-Risk (CoVaR) methodologies, this study provides a new perspective on understanding the asymmetry of systemic risk transmission between China and Asian emerging markets. Based on data from 2000 to 2024, the findings reveal significant spillover patterns, with Korea (KOSPI) showing high sensitivity to SSE risks, Malaysia (KLCI) exerting strong influence, and Thailand (SET) and Taiwan (TWII) emerging as key contributors and receivers of systemic risk. Under extreme market conditions, risk spillovers intensify, positioning SSE as a central hub in regional risk dynamics. These insights underscore the need for robust macroprudential policies and enhanced regional cooperation to mitigate systemic vulnerabilities, contributing to both the theoretical discourse on financial risk and its practical management.

2) Dataset selection: The rationale for market selection aligns with MSCI classifications, but the exclusion of certain regional markets should be more clearly justified.

A: With heartfelt thanks to the reviewers for their suggestions, we have added the following sections.

The Asian emerging markets selected for this study are based on MSCI's annual global market classification for 2024(https://www.msci.com/our-solutions/indexes/market-classification) and cover seven representative markets: India (BSE), Indonesia (JKSE), Taiwan (TWII), Malaysia (KLCI), the Philippines (PSI), Thailand (SET) and South Korea (KOSPI). These markets are defined by MSCI as Emerging Markets, characterized by high economic growth potential, developing capital markets, and increasing dependence on the global financial system. These markets have particularly strong geo-economic and financial ties to China, making them ideal for studying systemic risk spillovers between China and emerging markets in Asia. MSCI's market classification criteria not only consider economic scale and growth potential but also emphasize market liquidity, foreign investment accessibility, and the robustness of capital market infrastructure (Öner, 2022、Patel, 2021). Due to capital controls and insufficient market liquidity, countries such as Pakistan and Bangladesh are excluded from this study. Similarly, Vietnam is classified as a Frontier Market by MSCI, primarily due to its restrictions on foreign capital access and lower market liberalization, making it less suitable for assessing systemic risk spillovers in emerging markets.

Furthermore, this study focuses on systemic risk transmission between China and Asian emerging markets, which necessitates selecting markets with strong trade, investment, and capital flow linkages to China. The MSCI-classified Asian emerging markets are particularly sensitive to China's financial and economic fluctuations, making them more appropriate for analyzing China’s risk spillover effects in the region.

3) Risk Spillover Dynamics: While the study analyzes risk spillovers broadly, it would benefit from examining how risks propagate during specific financial crises. Although global events such as COVID-19 and the Russia-Ukraine war are mentioned, their specific impacts on systemic risk dynamics require more detailed exploration.

A: We sincerely appreciate the reviewer’s suggestion, we have incorporated Figure 2. Which presents a time-varying ΔCoVaR analysis to highlight the role of key economic and financial events in shaping systemic risk transmission.

Figure 2. Time-Varying ΔCoVaR between Emerging Markets and China’s Shanghai Stock Exchange (SSE)

Figure 2 illustrates that during the 2008 global financial crisis, ΔCoVaR values exhibited a sharp decline immediately after the Lehman Brothers bankruptcy, underscoring the impact of capital flow volatility on systemic risk (Acharya et al., 2012). Specifically, Thailand (SETI) and Taiwan (TWII) demonstrated high sensitivity to risk originating from the Chinese market, which can be attributed to their trade dependence on China and vulnerability to external shocks (Briguglio, 2016). Furthermore, the pronounced risk transmission from China’s SSE to South Korea (KOSPI) and Indonesia (JKSE) suggests that China progressively emerged as a regional risk hub during the crisis (Zhao & Park, 2024).

ΔCoVaR values during the COVID-19 outbreak indicate that supply chain disruptions and trade dependencies were primary drivers of systemic risk propagation. In the early stages of the pandemic (January to March 2020), significant risk spillovers flowed from Malaysia (KLCI) and Indonesia (JKSE) into the Chinese market. In contrast, later in the pandemic, risk spillovers from China’s SSE intensified toward export-oriented economies such as South Korea (KOSPI) and Taiwan (TWII) (Ling et al., 2023).

ΔCoVaR results from the Russia-Ukraine war indicate that, while the war’s immediate impact was concentrated in energy and commodities markets, its financial repercussions extended to Asian economies via trade uncertainty and supply chain disruptions (García-Herrero & Mu, 2023). Notably, the ΔCoVaR values for Taiwan (TWII) and South Korea (KOSPI) increased markedly during the initial years of the conflict, underscoring these markets' heightened susceptibility to energy price volatility and geopolitical uncertainty. Additionally, variations in the ΔCoVaR time series reveal significant asymmetries in systemic risk transmission. For instance, risk spillovers from China’s SSE to Taiwan and South Korea were notably stronger than the reverse, a pattern primarily driven by regional differences in trade structures and capital flows. Conversely, Malaysia and Thailand exhibited higher ΔCoVaR values for China, indicating a stronger reliance on the Chinese market during crises.

From a historical standpoint, during the 2000 dot-com bubble collapse, China was not yet fully integrated into the global financial system, resulting in limited risk spillovers to South Korea (KOSPI) and Taiwan (TWII). However, Indonesia (JKSE) and Malaysia (KLCI) had greater risk inputs into the Chinese market due to shifts in global trade dynamics (Zhou & Tyers, 2022). Following the 2015 Chinese stock market crisis, China’s SSE played an increasingly dominant role in systemic risk transmission, with spillovers to Taiwan and South Korea expanding significantly. ΔCoVaR results further suggest that China transitioned from a ‘risk taker’ to a ‘risk transmitter’ as its financial market integration deepened (Chen et al., 2023). This period also exhibited a clear asymmetry in systemic risk spillovers, with China exporting significantly more risk to neighboring markets than it absorbed in return.

4) Literature and Emerging Markets: The literature review could be expanded to include recent studies on systemic risk and financial contagion, particularly in emerging markets.

A: Sincere thanks to the reviewers for their valuable feedback, which helped us refine the literature review to provide a more in-depth and critical discussion. The following revisions have been added to the literature review to address the specific issues raised:

The literature on financial market spillovers has evolved significantly, integrating theoretical and empirical advancements. Beyond mean and volatility spillovers, recent studies emphasize alternative risk measures for systemic risk transmission. Diebold and Yilmaz (2014) developed a network topology model using directed acyclic graphs to capture financial market interconnectedness, identifying key systemic nodes in risk propagation. Similarly, Baruník and Křehlík (2018) introduced a frequency dynamics spillover approach, distinguishing between short- and long-term spillover effects, providing deeper insights into market shock transmission mechanisms. These advanced econometric models highlight the need for non-traditional approaches in financial risk assessment.

2.3 Empirical Findings on Asian Markets

An increasing body of research explores the role of macroeconomic factors in shaping financial market spillovers. Kannadhasan and Das (2020) analyzed and compared the impact of economic policy uncertainty (EPU) and geopolitical risk (GPR)-related shocks on emerging Asian equity markets. Their findings suggest that increased uncertainty amplifies spillover effects, particularly in emerging markets, which are more exposed to external shocks. Monetary policy decisions play a crucial role—Ehrmann and Fratzscher (2005) showed that central bank interest rate changes, especially by the Federal Reserve, trigger global financial market reactions, affecting capital flows and asset price volatility. Gagnon et al. (2011) further demonstrated that unconventional monetary policies, such as quantitative easing, heighten financial integration and increase spillover intensity across international markets.

Recent studies highlight the unique vulnerabilities and transmission mechanisms of financial spillovers in emerging markets. These markets tend to exhibit heightened sensitivity to global financial disruptions due to capital flow volatility, regulatory constraints, and limited market depth. For example, Sula (2010) found that emerging economies experience stronger contagion effects during global financial instability, particularly when capital flight and exchange rate pressures increase. Similarly, Azad and Serletis (2020) demonstrated that U.S. monetary policy spillovers disproportionately impact emerging markets under conditions of heightened uncertainty.

Khawaja (2021) emphasized emerging markets’ growing importance in global financial networks due to their rapid economic growth, expanding capital markets, and increasing foreign investment. However, their structural fragility, as discussed by Karanasos et al. (2022), amplifies systemic risk compared to developed economies. Akyüz (2017) found that the structure of commodity trade in commodity-dependent developing countries plays a significant role in shaping their financial vulnerability. Additionally, Wang and Wang (2024) analyzed the interactions between BRICS stock markets and cryptocurrencies, finding increased financial interdependencies during market turbulence. Moreover, most Asian equity and foreign exchange markets are net risk receivers (Wang et al., 2023). Feng et al., (2023) study the systematic risk spillovers from the Belt and Road equity markets between 2008 and 2021, and analyse that the network effects are asymmetric and regionally heterogeneous across markets at different risk levels.

2.4 Limitations of Previous Studies

Despite the recognition of systemic risk and financial contagion in emerging markets, most existing studies focus on developed markets or rely on traditional econometric techniques that assume linear dependence. While some recent studies (e.g., Wang et al., 2023) have explored quantile-based spillovers, research specific to Asian emerging markets remains limited, and the dynamics of risk spillovers under extreme market conditions require further exploration. Given Asia’s increasing financial integration and evolving market structure, a more refined understanding of risk spillover dynamics is essential.

Moreover, existing spillover models often fail to fully capture risk transmission under extreme market conditions. Traditional models such as VAR and GARCH can effectively model mean spillovers but lack the granularity needed to assess tail risk and asymmetric dependence. Most prior research typically treats systemic risk spillovers as mutually reinforcing relationships, without analyzing whether one market acts as a dominant risk transmitter while others function as net receivers.

To address these gaps, this study employs quantile regression and CoVaR methods to distinguish between China’s impact on Asian emerging markets and the influence of Asian markets on China. This approach provides a detailed understanding of asymmetric systemic risk propagation, highlighting the directionality and magnitude of risk transmission between markets. By focusing on these interactions, this study aims to provide new insights into Asia’s role in global financial stability and to inform policymakers in developing tailored risk management strategies in an increasingly interconnected world.

5) Visual Aids: Incorporating visual aids like influence networks to illustrate risk spillovers would improve the clarity and accessibility of the findings.

A: We sincerely appreciate the reviewer’s suggestion to construct a CoVaR-based network to enhance the systemic perspective of risk spillover effects. In response to this recommendation, we have incorporated Figure 1, which provides a network analysis of systemic risk contributions using two key metrics: (the absolute difference in systemic risk contribution (ΔCoVaR^SSE - ΔCoVaR^i) and the relative difference in systemic risk contribution (%CoVaR^SSE - %CoVaR^i). And deleted the previous fig1 and fig2, and re-added Figure 2. Time-Varying ΔCoVaR between Emerging Markets and China’s Shanghai Stock Exchange (SSE)

Figure 1. Network Analysis of Systemic Risk Contributions: Absolute vs. Relative Perspectives

To further analyze the directionality and asymmetry of systemic risk transmission, this paper employs two key metrics: the absolute difference in systemic risk contribution (ΔCoVaR^SSE - ΔCoVaR^i) and the relative difference in systemic risk contribution (%CoVaR^SSE - %CoVaR^i). The corresponding results are presented in Figure 1.

The absolute difference metric (ΔCoVaR^SSE - ΔCoVaR^i) quantifies the net impact of systemic risk transmission between SSE and other markets, making it particularly useful for assessing how systemic risk propagates from individual markets to SSE. Negative values (red arrows) indicate that a market contributes more systemic risk to SSE, classifying it as a ‘Risk Contributor,’ meaning SSE is more affected by it. Examples include KLCI (-0.209), SET (-0.199), PSI (-0.069), and TWII (-0.035). Conversely, positive values (blue arrows) indicate that SSE contributes more to the systemic risk of a given market, classifying it as a ‘Risk Receiver,’ meaning the market is more susceptible to SSE’s risk transmission. Examples include KOSPI (0.143), BSE (0.099), and JKSE (0.016).

In contrast, the relative difference metric (%CoVaR^SSE - %CoVaR^i) normalizes ΔCoVaR to capture the relative strength of systemic risk propagation, making it particularly suitable for cross-market comparisons. Negative values (red arrows) indicate that a market contributes more systemic risk to SSE in relative terms, classifying it as a ‘Relative Risk Contributor.’ For instance, KOSPI (-4.239%), BSE (-6.049%), JKSE (-5.496%), and KLCI (-1.860%) exert stronger systemic risk shocks on SSE during systemic events. Positive values (blue arrows) indicate that SSE contributes more systemic risk to a market in relative terms, classifying it as a ‘Relative Risk Receiver.’ This is observed in SET (8.539%), PSI (0.361%), and TWII (0.299%), suggesting that SSE’s market volatility has a more pronounced impact on these markets.

It is crucial to note that while ΔCoVaR quantifies the absolute magnitude of systemic risk contribution, %CoVaR captures the asymmetry in risk transmission pathways. By integrating these two indicators, we can gain a more comprehensive understanding of both the directional flow of systemic risk and the asymmetric nature of its propagation across markets, particularly in the aftermath of major uncertainties.

6) Policy Recommendations: While the study suggests strengthening macroprudential frameworks, the recommendations are generic. The findings should be used to propose specific policies to mitigate systemic vulnerabilities in the region.

A: We sincerely appreciate the reviewer’s insightful feedback regarding the policy recommendations. In response, we have expanded and refined this section to propose specific, actionable policies that align with the study’s empirical findings. The revised policy recommendations are categorized into two key areas in Conclusion :

Policy recommendations based on strengthening systemic risk management and market resilience building can be divided into the following two parts:

1) Enhancing systemic risk monitoring and regional financial stability mechanisms. This study finds that Korea (KOSPI) and Taiwan (TWII) are the most sensitive to market risk in China (SSE), while Malaysia (KLCI) and Thailand (SET) have the greatest impact on systemic risk in SSE. This phenomenon suggests that there are complex spillover effects of systemic risk in Asian emerging markets, and a more comprehensive regional financial risk monitoring and stabilisation mechanism needs to be established to enhance overall financial resilience (Stanciu & Spulbăr, 2024). For example, it is recommended to establish an ‘Asian financial systemic risk monitoring network’, integrate the data resources of financial regulators of various countries, and build a cross-border financial risk early-warning system using big data and artificial intelligence technology, so as to track market fluctuations in real time and identify the path of systemic risk contagion. In addition, financial data sharing and cross-border regulatory cooperation among Asian countries should be promoted, especially in the areas of capital flows, institutional leverage levels, and financial derivatives trading, so as to enhance the ability to identify market risks and avoid the accumulation of systemic risks.

At the same time, it is recommended to make use of the multilateral cooperation frameworks of “BRICS and the Belt and Road Initiative (BRI)” to deepen regional financial market collaboration, enhance financial infrastructure and reduce market volatility. Financial cooperation under the BRICS New Development Bank (NDB) and the Belt and Road Initiative (BRI) can serve as an important pillar of regional financial stability, promote financial regulatory coordination among relevant countries/regions, and improve the defence capability against global capital market turbulence.

2) Optimising capital buffers, controlling cross-border capital flows and promoting market diversification

The high degree of interconnectedness of Asian emerging markets dictates that financial risks are not confined to a single market, but are transmitted through multiple channels such as capital flows, investor behaviour, exchange rate volatility, etc. (Aftab & Mehmood, 2023). Therefore, the macroprudential management framework should be strengthened to optimise the capital buffer mechanism, while promoting capital market diversification to reduce the risk of market homogeneity.

In terms of capital management, South Korea (KOSPI) and Taiwan (TWII), as the most sensitive markets to SSE risk, need to strengthen the capital buffer mechanism in the banking and securities markets, and increase the capital adequacy requirement to enhance the ability to cope with SSE market volatility. In addition, for markets with strong risk transmission, such as Malaysia (KLCI) and Thailand (SET), it is recommended to introduce macro-prudential capital flow management tools (e.g., short-term capital flow tax, minimum holding period for capital, etc.) to avoid short-term capital outflows from exacerbating market turbulence in times of severe market volatility. Meanwhile, in terms of market diversification construction, countries should promote the synergistic development of bond markets and stock markets to reduce the systemic shock caused by a single market crash. In addition, regulators can promote institutional investors (e.g., pension funds, insurance companies) to expand their positions in asset classes such as bonds, alternative investments, and commodities to reduce the impact of stock market volatility on the overall financial market.

Finally, it is recommended to use fintech to enhance market resilience by promoting AI-driven market risk monitoring systems combined with smart melting mechanisms to improve market stability (Hamdan & Braendle, 2024). Under extreme market stress, intelligent trading restrictions can be implemented to prevent liquidity crises from occurring through automated liquidity support tools that inject funds when market liquidity is tight.

In summary, emerging markets in Asia should reduce the contagion effect of cross-border financial risks, improve overall market resilience and promote Strengthened regional financial cooperation, such as information sharing and coordinated risk management strategies, can also play a pivotal role in mitigating systemic risks (Forbes & Rigobon, 2002).

7) Typographical Errors: The paper includes typographical errors, such as inconsistencies in notations. For instance, the price at day $t$ is referred to as $R_{t}$ in the text but is denoted as $P_{t}$ in the equations.

$P_{t}$ and $P_{t-1}$ are the closing prices of stocks on day $t$ and $t-1$, respectively:

$R_{t}=100\times Ln(\frac{P_{t}}{P_{t-1}})$	(9)
